# Sub-generator fat as a potential risk factor for high defibrillation threshold in an extravascular implantable cardioverter-defibrillator: A case report

**DOI:** 10.1016/j.hrcr.2026.02.017

**Published:** 2026-02-27

**Authors:** Tsukasa Oshima, Kenichiro Yamagata, Fujiu Katsuhito, Norihiko Takeda

**Affiliations:** Department of Cardiovascular Medicine, Graduate School of Medicine, The University of Tokyo, Tokyo, Japan

**Keywords:** Extravascular implantable cardioverter-defibrillator, Defibrillation threshold, Sub-generator fat, Fat removal, Impedance

**Key Teaching Points**

• In subcutaneous implantable cardioverter defibrillators (ICD), sub-generator fat is a known risk factor for high defibrillation threshold (DFT). However, its impact on DFT in extravascular ICD (EV-ICD) systems has not been well described.
• We report a case of high DFT during EV-ICD implantation, in which sub-generator fat was associated with elevated impedance and defibrillation failure, with improvement after fat removal.
• Sub-generator fat may also represent a modifiable risk factor for high DFT in EV-ICD systems.

## Introduction

Implantable cardioverter-defibrillators (ICDs) effectively prevent sudden cardiac death.^1^ The extravascular ICD (EV-ICD) is a novel system that avoids transvenous leads by placing a single lead in the substernal space.^2^^,^^3^ Although defibrillation threshold (DFT) testing is typically recommended for EV-ICDs, the factors associated with high DFT remain unclear. In subcutaneous ICDs (S-ICDs), sub-coil and sub-generator fat have been reported as predictors of high DFT, likely because of attenuation of the shock vector.^4^ It is not known whether a similar effect occurs in EV-ICD systems. We present a case in which a substantial fat layer beneath the generator was associated with elevated high-voltage impedance and DFT failure. This highlights the potential impact of sub-generator fat on the efficacy of EV-ICD defibrillation.

## Case report

A 41-year-old man with dilated cardiomyopathy was admitted for the implantation of an EV-ICD as a primary prevention of sudden cardiac death. His height was 167 cm, weight 88.0 kg, and body mass index (BMI) 31.6 kg/m^2^.

**Figure 1:**
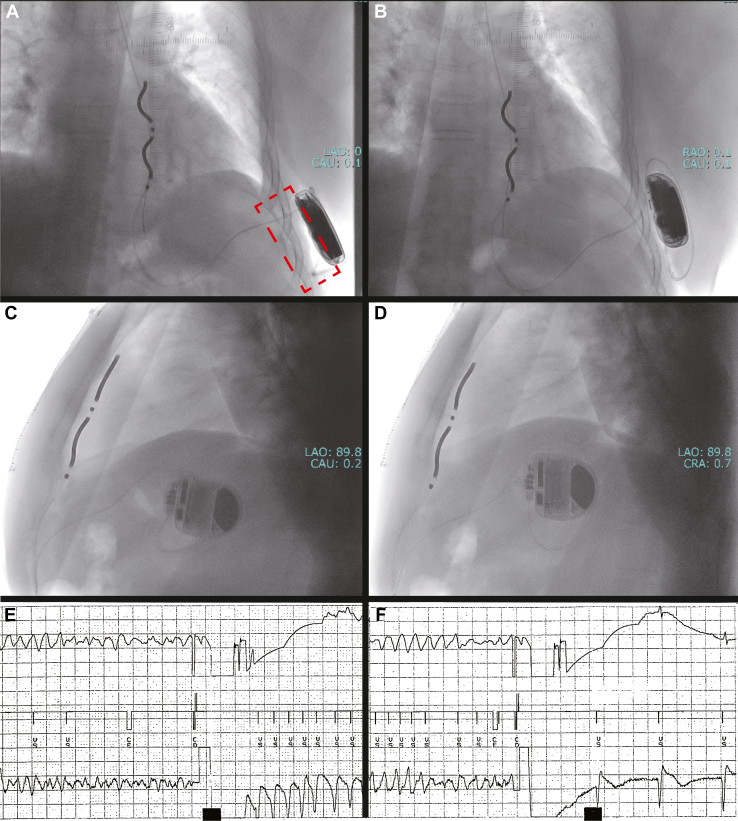
**A and B:** Antero-posterior views of an extravascular implantable cardioverter defibrillator (EV-ICD) before **(A)** and after **(B)** fat removal. The *red square* indicates the presence of fat beneath the generator. **C and D:** Lateral views of EV-ICD before **(C)** and after **(D)** the fat removal. **E and F:** Electrocardiograms of EV-ICD before **(E)** and after **(F)** the fat removal. The first defibrillation threshold (DFT) testing failed **(E)**, but ventricular fibrillation was successfully terminated by a 30-J shock after the fat was eliminated **(F)**.

The procedure was performed under general anesthesia in a hybrid operation room. The substernal lead was successfully inserted on the first attempt. The R-wave amplitude between ring 1 and ring 2 was 1.5 mV without P-wave sensing. The generator was placed in the approximately recommended left mid-axillary position (Figure 1A and 1B). However, a substantial layer of fat was present beneath the generator (Figure 1A). After connecting the lead to the generator, the generator was secured with sutures incorporating the surrounding fat and underlying fascial layer to ensure stability, and DFT testing was performed. Although ventricular fibrillation (VF) was induced, undersensing prevented automatic shock delivery. A manually delivered 30-J shock that subsequently also failed to terminate VF (Figure 1C), requiring an external 270-J shock. High-voltage (HV) impedance at this time was 100 Ω.

As the fat layer beneath the generator was considered a potential contributor to the elevated impedance, the generator pocket was revised, and the sub-generator fat was completely removed. The generator was then repositioned at nearly the same anatomical location (Figure 1D, E). Sensing settings were adjusted to avoid undersensing, from 0.45 mV to 0.20 mV, and repeat DFT testing was performed. VF was successfully detected, and a 30-J shock promptly restored sinus rhythm (Figure 1F). At this point, HV impedance decreased to 81 Ω. The patient recovered uneventfully and was discharged without complication.

## Discussion

In this case, substantial fat beneath the generator pocket was temporally associated with elevated HV impedance and failure of a 30-J shock to terminate induced VF. Following the removal of the sub-generator fat, the HV impedance decreased, and a subsequent 30-J shock successfully restored sinus rhythm. These observations suggest that sub-generator fat can negatively affect EV-ICD defibrillation performance and may contribute to high DFT.

Fat is electrically less conductive than muscle or blood, and can therefore increase the impedance along the shock pathway, reducing the amount of energy that reaches the myocardium.^4^ Increased impedance reduces current delivery for a given energy setting and can alter the effective shock vector, both of which may increase the DFT or cause shock failure. This biophysical rationale has been proposed to explain similar findings in S-ICDs, where sub-coil and sub-generator fat have been identified as risk factors for higher DFTs. Given that EV-ICD generators are placed in a subcutaneous pocket and the lead runs substernally, fat beneath the generator may similarly increase impedance, and lead to DFT testing failure.

A recent report by Gutierrez et al^5^ described 2 patients with a BMI >45 kg/m^2^ who underwent EV-ICD implantation and successfully passed DFT testing. In both cases, the generator was positioned with minimal subcutaneous fat beneath the generator, based on the published figures. Although these cases demonstrate that obesity alone does not necessarily lead to elevated DFT in EV-ICD systems, they also support the hypothesis that the distribution of fat, particularly sub-generator fat, may be more relevant than BMI itself.

Our case is consistent with published impedance data. For example, in the Acute Extravascular Defibrillation, Pacing and Electrogram study, the average impedance was 91.7 ± 22.0 Ω and 107.5 ± 31.8 Ω among subjects with success or failure of a 30-J shock, respectively.^6^^,^^7^ In another study, the average impedance was 66.7 ± 10.3 and 78.0 ± 1.4 Ω among subjects with success or failure of a 30-J shock, respectively.^2^^,^^7^ In both studies, the observed average impedance was lower in subjects who experienced success than in those who experienced failure, and the differences of impedance are similar to that observed in our case before and after the fat removal.

In S-ICDs, the position of the generator, as quantified by the Prospective Randomized Comparison of Subcutaneous and Transvenous Implantable Cardioverter-Defibrillator Therapy score, has also been shown to influence defibrillation efficacy.^8^ However, an equivalent scoring system or optimal generator position for EV-ICDs has yet been established. In our case, the generator was repositioned to a similar anatomical location, suggesting that the improvement in defibrillation efficacy was primarily because of the reduction in sub-generator fat rather than a change in generator position.

Our observations indicate that sub-generator fat may increase HV impedance and contribute to DFT failure in EV-ICD. During EV-ICD pocket creation, surgeons should pay attention to the thickness of the sub-generator fat and consider removing fat when a large subcutaneous layer is present, particularly in obese patients. As a limitation of our case, since air and residual sub-generator fat could not be clearly distinguished on fluoroscopy, the independent contribution of air to the elevated impedance and DFT remains uncertain. Further studies are warranted to evaluate the impact of sub-generator fat on DFT testing in EV-ICDs.

## Disclosures

The authors have no conflicts of interest to disclose.
